# Methodological Approach for Optogenetic Manipulation of Neonatal Neuronal Networks

**DOI:** 10.3389/fncel.2017.00239

**Published:** 2017-08-14

**Authors:** Sebastian H. Bitzenhofer, Joachim Ahlbeck, Ileana L. Hanganu-Opatz

**Affiliations:** Developmental Neurophysiology, Institute of Neuroanatomy, University Medical Center Hamburg-Eppendorf Hamburg, Germany

**Keywords:** development, optogenetics, *in utero* electroporation, prefrontal cortex, hippocampus

## Abstract

Coordinated patterns of electrical activity are critical for the functional maturation of neuronal networks, yet their interrogation has proven difficult in the developing brain. Optogenetic manipulations strongly contributed to the mechanistic understanding of network activation in the adult brain, but difficulties to specifically and reliably express opsins at neonatal age hampered similar interrogation of developing circuits. Here, we introduce a protocol that enables to control the activity of specific neuronal populations by light, starting from early postnatal development. We show that brain area-, layer- and cell type-specific expression of opsins by *in utero* electroporation (IUE), as exemplified for the medial prefrontal cortex (PFC) and hippocampus (HP), permits the manipulation of neuronal activity *in vitro* and *in vivo*. Both individual and population responses to different patterns of light stimulation are monitored by extracellular multi-site recordings in the medial PFC of neonatal mice. The expression of opsins via IUE provides a flexible approach to disentangle the cellular mechanism underlying early rhythmic network activity, and to elucidate the role of early neuronal activity for brain maturation, as well as its contribution to neurodevelopmental disorders.

## Introduction

Specific patterns of rhythmic network activity synchronize neuronal networks during early brain development (Hanganu et al., 2006; Khazipov and Luhmann, 2006; Brockmann et al., 2011). Together with genetic programs this electrical activity controls the maturation of neuronal networks (Khazipov and Luhmann, 2006; Hanganu-Opatz, 2010). The initial development of neuronal networks is controlled by molecular cues and largely independent of evoked neurotransmitter release (Molnár et al., 2002; Washbourne et al., 2002). However, subsequently diverse developmental processes such as neuronal migration, differentiation, axon growth, synapse formation, programmed cell death, and myelination are influenced by neuronal activity and mediate the activity-dependent maturation of neuronal networks (Spitzer, 2002; Heck et al., 2008; De Marco García et al., 2011; Kirkby et al., 2013; Mitew et al., 2016). Spontaneous and sensory-triggered discontinuous patterns of oscillatory activity (e.g., spindle bursts, nested gamma spindle bursts, beta and gamma oscillations) have been shown to influence the maturation of cortical and cortico-subcortical networks (Hanganu-Opatz, 2010). However, the cellular mechanisms generating the different patterns of early network activity are still largely unknown. Furthermore, direct evidence for the impact of early activity on the maturation of neuronal networks is still missing.

Specific contributions of distinct neuronal populations to rhythmic network activity in the adult brain and their influence on behavior have been resolved during the last decade using optogenetics approaches (Cardin et al., 2009; Adesnik and Scanziani, 2010). Selective expression of light sensitive membrane channels and pumps in defined neuronal populations allow for precisely timed control of the activity of these neurons in intact networks (Fenno et al., 2011). The optogenetic approach helped to interrogate a large diversity of neural circuits in the adult brain involved in sensory processing (Lepousez and Lledo, 2013; Olcese et al., 2013), memory (Liu et al., 2013; Johansen et al., 2014; Spellman et al., 2015) and neuropsychiatric disorders (Tye and Deisseroth, 2012; Steinberg et al., 2015).

Similar application of optogenetics in the developing brain has been hampered by the lack of flexible methods to selectively and effectively target neurons at early age. The most common strategies to express light-sensitive proteins in the adult rodent brain are viral transduction and genetically modified mouse lines (Zhang et al., 2010; Yizhar et al., 2011). Both techniques require cell-type specific promoters to restrict the expression to a neuronal subpopulation. Most promoters have been shown to undergo qualitative and quantitative transitions during development that can lead to unspecific and unstable expression (Sánchez et al., 1992; Kwakowsky et al., 2007; Wang et al., 2013). While recently synapsin has been successfully used as promoter for viral injections in neonatal rats and led to reliable activation of neurons in the visual cortex (Murata and Colonnese, 2016), most promoters that specifically label neuronal subpopulations yield only little expression during development. Thereby, viral transduction is only of limited usability to investigate local network interactions during development. Furthermore, most viruses require 10–14 days until reliable and sufficient expression is achieved, too long for the interrogation of neonatal networks. On the other hand, recently engineered viruses yielding fast expression are often toxic to the expressing cells even after short time periods (Klein et al., 2006), limiting their applicability for long-term investigations.

Another strategy for controlling the activity of developing circuits relies on genetically modified mouse lines. By these means the activity of gamma-aminobutyric acid (GABA)ergic interneurons was controlled by light during early postnatal development using the glutamic acid decarboxylase promoter (Valeeva et al., 2016). However, the major drawback of this approach is the lack of area specificity, the light-sensitive opsins being expressed in the entire brain. Attempts to spatially confine the illumination are useful, but cannot avoid that long range projections are co-activated and interfere with the investigation of the area of interest.

Area and cell-type specific transfection of neurons without the need of specific promoters has been achieved by *in utero* electroporation (IUE). This technique, mainly used to investigate neuronal migration during embryonic development, enables targeting of precursor cells of neuronal and glial subpopulations, based on their distinct spatial and temporal patterns of generation in the ventricular zone (Tabata and Nakajima, 2001; Borrell et al., 2005; Niwa et al., 2010; Hoerder-Suabedissen and Molnar, 2015). This makes IUE the method of choice for the selective targeting of neuronal populations during development. In combination with optogenetics and electrophysiology *in vivo* it enables the interrogation of developing circuits and the elucidation of initial cortical wiring. By these means, we recently elucidated the role of pyramidal neurons in layers II/III and V/VI of the medial prefrontal cortex (PFC) for the generation of frequency-tuned patterns of oscillatory activity (Bitzenhofer et al., 2017).

Here, we introduce the protocol for area-, layer- and cell-type specific manipulation of neurons by light throughout postnatal development in mice and exemplify it for the PFC or hippocampus (HP). We illustrate for the PFC that site-directed IUE of highly efficient opsins under control of a ubiquitous promoter yields sufficient population-specific expression to trigger action potentials and rhythmic network activity during early development *in vitro* and *in vivo*.

## Materials and Methods

All experiments were performed in compliance with the German laws and the guidelines of the European Community for the use of animals in research and were approved by the local ethical committee (Behörde für Gesundheit und Verbraucherschutz/Lebensmittelsicherheit und Veterinärwesen; 111/12, 132/12).

### Area-, Layer- and Cell Type-Specific Expression at Neonatal Age by *In Utero* Electroporation

Pregnant C57Bl/6J mice were received from the animal facility of the University Medical Center Hamburg-Eppendorf at 6 days of gestation, housed individually in breeding cages at a 12 h light/dark cycle, and fed *ad libitum*. The day of vaginal plug detection was defined as embryonic day (E) 0.5. Additional wet food was provided on a daily basis from E6.5 and was supplemented with two drops of Metacam (0.5 mg/ml, Boehringer-Ingelheim, Germany) from 1 day before until 2 days after IUE.

**Figure 1:**
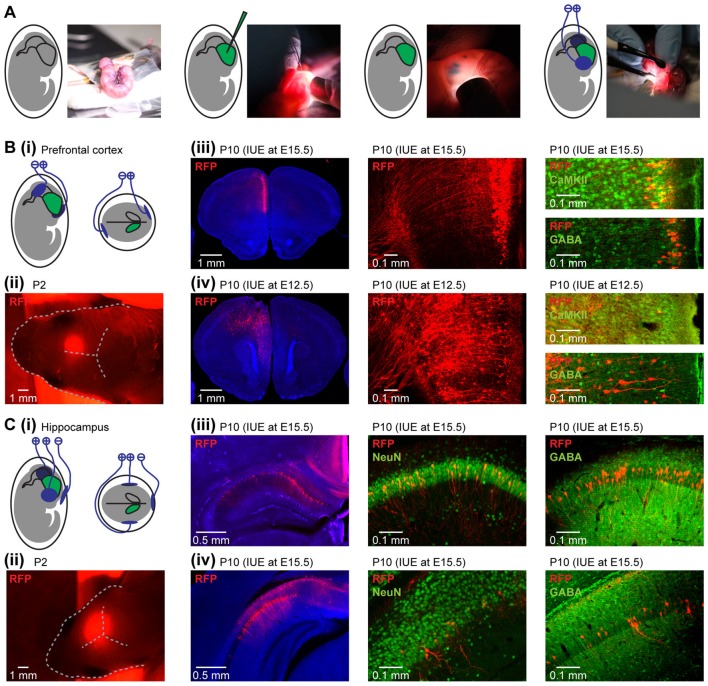
Area-, layer- and cell-type-specificity of neuronal targeting by *in utero* electroporation (IUE). **(A)** Diagrams and photographs illustrating the procedure for IUE. **(Bi)** Schematic representation of the position of electroporation paddles to yield expression in pyramidal neurons in the medial prefrontal cortex (PFC). **(ii)** Photograph showing the expression of red fluorescent protein (RFP; red) transfected in the PFC by IUE detectable through skull and skin at P2. **(iii)** Targeting of pyramidal neurons in layer II/III of the neonatal PFC. Left, RFP expressing cells (red) in 50 μm-thick coronal slices of a P10 mouse at the level of the PFC after IUE at E15.5. Middle, RFP-expressing neurons in prefrontal layer II/III displayed at higher magnification. Right, confocal images of RFP-expressing neurons after staining for Ca2+/calmodulin-dependent protein kinase II (CaMKII) or gamma-aminobutyric acid (GABA). **(iv)** Targeting of pyramidal neurons in layer V/VI of the neonatal PFC. Same as **(iii)** for IUE at E12.5. **(Ci)** Schematic representation of the position of the electroporation paddles to yield expression in pyramidal neurons of hippocampal CA1 area. **(ii)** Photograph showing the expression of RFP (red) transfected in the hippocampus (HP) by IUE detectable through skull and skin at P2. **(iii)** Targeting of pyramidal neurons in dorsal hippocampus (HP). Left, RFP expressing cells (red) in 50 μm-thick coronal slices of a P10 mouse at the level of the dorsal HP after IUE at E15.5. Middle, RFP-expressing neurons in dorsal HP displayed at higher magnification after staining for NeuN. Right, confocal images of RFP-expressing neurons after staining for GABA. **(iv)** Same as **(iii)** for the intermediate HP.

At E12.5 or E15.5 pregnant dams were injected subcutaneously with buprenorphine (0.05 mg/kg body weight) 30 min before surgery. Surgery was performed under isoflurane anesthesia (induction: 5%, maintenance: 3.5%) on a heating blanket. The eyes of the dam were covered with ointment to prevent damage. The abdomen was shaved, without causing damage to the nipples, and wiped with an ethanol pad. The dam was positioned on its back on a sterile mat and covered with parafilm sparing the abdomen. Toe pinch reflex and breathing were monitored throughout the surgery. The abdomen was sterilized with povidone-iodine solution (100 mg/ml). A 10 mm long incision along the midline was made successively through the abdominal skin and muscle layer. Uterine horns were carefully exposed using ring forceps. Throughout surgery, uterine horns were kept moist with warm sterile phosphate buffered saline (PBS, 37°C). Pulled borosilicate glass capillaries with a sharp and long tip were used to inject 0.75–1.25 μl solution containing 1.25 μg/μl plasmid DNA coding for a channelrhodopsin 2 mutant and red fluorescent protein (RFP), or just the fluorescent reporter (pAAV-cytomegalovirus enhancer fused to chicken beta-actin (CAG)-tDimer2 or pAAV-CAG-channelrhodopsin 2 double mutant E123T T159C (ChR2(ET/TC))-2A-tDimer2) into the right lateral ventricle of each embryo. The ubiquitous CAG promoter was used to achieve robust expression. 0.1% fast green dye was added to the solution for visual control of the injection site. Bright illumination helps to detect the lateral ventricles of the embryos (Figure 1A).

Depending on the orientation of the electroporation paddles, selective transfection of a subset of neuronal precursor cells in the ventricular zone by IUE yielded area-specific expression of light-sensitive proteins. For targeting PFC, the embryo’s head was placed between the bipolar electroporation tweezer-type paddles (3 mm diameter for E12.5, 5 mm diameter for E15.5, Protech, TX, USA) at a 20° leftward angle from the midline and a 10° angle downward from anterior to posterior with the positive pole on the hemisphere contralateral to the injection (Figure 1Bi). Voltage pulses (35 V, 50 ms) were applied five times at intervals of 950 ms controlled by an electroporator (CU21EX, BEX, Japan) to target the transfection of precursor cells of glutamatergic neurons in the specific area of the subventricular zone that migrate into the medial PFC. For targeting HP, tripolar electroporation paddles were used at E15.5 (dal Maschio et al., 2012; Szczurkowska et al., 2016) and the embryo was placed between the electroporation tweezer-type paddles (5 mm diameter, both positive poles, Protech, TX, USA). A third custom build negative pole was positioned on top of the head roughly between the eyes (Figure 1Ci). Voltage pulses (30 V, 50 ms) were applied six times at intervals of 950 ms. Warm sterile PBS was applied to the embryo’s head immediately after the voltage pulses.

Uterine horns were placed back into the abdominal cavity after electroporation. During the entire procedure, damage to the blood vessels surrounding the uterine horns was avoided. The abdominal cavity was filled with warm sterile PBS and the abdominal muscles and skin were sutured individually with absorbable and non-absorbable suture thread, respectively. After recovery from anesthesia, pregnant mice were returned to their home cages, which were half placed on a heating blanket for 2 days after surgery. After IUE, the recovery of the dam was visually inspected every day and special attention was given to minimize the stress level. The day of birth was defined as postnatal day (P) 0. IUE can be performed by a single person. However, the quality of the surgery can be significantly improved by reducing its duration if a second person assists in positioning the embryos for the construct injections. Surgeries of short duration are desirable, since they improve the survival rate of IUE-manipulated pups.

Fluorophore-expression in the area of interest can be detected through the intact skin and skull with a fluorescent flash light (Nightsea, MA, USA) or a standard fluorescence microscope until the age P2 (Figures 1Bii,Cii). To confirm area-, layer- and cell type-specific transfection, pups of *in utero* electroporated dams were anesthetized with 10% ketamine (aniMedica, Germany)/2% xylazine (WDT, Germany) in 0.9% NaCl solution (10 μg/g body weight, i.p.) and transcardially perfused with Histofix (Carl Roth, Germany) containing 4% paraformaldehyde. Brains were removed, postfixed in 4% paraformaldehyde for 24 h, sectioned coronally at 50 μm, and stored at 4°C in the dark in sterile PBS containing 0.05% sodium azide. The resulting slices were transferred to glass slides and covered with Fluoromount (Sigma-Aldrich, St. Louis, MO, USA). Wide field fluorescence images were acquired to localize tDimer2 expression after IUE.

Immunohistochemical stainings were performed to confirm cell type-specific expression. Free-floating slices were permeabilized and blocked in PBS containing 0.8% Triton X 100 (Sigma-Aldrich, St. Louis, MO, USA), 5% normal bovine serum (Jackson Immuno Research, West Grove, PA, USA) and 0.05% sodium azide. Slices were washed and incubated on a shaker at 4°C for 24 h with mouse monoclonal Alexa Fluor-488 conjugated antibody against NeuN (1:200, MAB377X, Merck Millipore, MA, USA), rabbit polyclonal primary antibody against Ca^2+^/calmodulin-dependent protein kinase II (CaMKII; 1:200, PA5-38239, Thermo Fisher Scientific, MA, USA), or rabbit polyclonal primary antibody against GABA (1:1000, #A2052, Sigma-Aldrich, St. Louis, MO, USA). For CaMKII and GABA, after washing, slices were incubated at room temperature for 2 h with Alexa Fluor-488 goat anti-rabbit IgG secondary antibody (1:500, A11008, Merck Millipore, MA, USA). Slices were transferred to glass slides and covered with Fluoromount (Sigma-Aldrich, MO, USA). Images were acquired with a confocal microscope (DM IRBE, Leica, Germany) to quantify tDimer2 expression and to analyze immunohistochemical stainings.

For the PFC, layer-specific targeting of neurons was obtained by conducting the IUE at distinct embryonic stages. IUE at E15.5 leads to expression in superficial layers of the medial PFC (Figure 1Biii), whereas the same protocol at E12.5 results in expression in layers V/VI (Figure 1Biv), due to the subsequent formation of cortical layers in an inside-out sequence. Prefrontal expression was achieved in about 80% of pups electroporated at E15.5 and in about 40% of pups electroporated at E12.5. The lower success rate for IUE at E12.5 is most likely due to the smaller size of the embryos. Co-transfection of nearby cortical regions was present in about 10% of animals when targeting PFC. The size of the expression area can be modified by the size of the electroporation paddles. Paddles with a diameter of 5 mm at E15.5 or 3 mm at E12.5 led to transfection 400–600 μm along the anterior-posterior axis in the PFC. The distinct origin of glutamatergic and GABAergic neurons allows for cell-type-specific transfections. The protocols described above led to targeting of glutamatergic neurons with the fraction of transfected cells varying between 20% and 40%. While IUE at E15.5 resulted in almost exclusive labeling of neurons in superficial cortical layers (99.3 ± 0.2% in layer II/III), IUE at E12.5 had poorer layer specificity (87.7 ± 0.9 in layer V/VI), due to an overlap in the generation of cortical neurons in different layers from the same progenitor cells (Martínez-Garay et al., 2016). The strong intensity of the RFP tDimer2 and its distribution throughout the entire neuron enabled to detect dendritic morphology and axonal projections of expressing neurons (Figures 1Biii,iv). Furthermore, expression was stable during the entire developmental period. IUE with tripolar electroporation paddles at E15.5 yielded expression in the pyramidal layer of the HP in about 60% of newborn pups (Figure 1Ci). Weak co-transfection of neurons in the retrosplenial cortex was detected in all investigated pups. The fluorophore was detectable in the HP through the skin and skull until P2 (Figure 1Cii). The IUE configuration yielded expression in the dorsal (Figure 1Ciii) and intermediate HP (Figure 1Civ). Similar to the PFC, transfection in the HP was restricted to glutamatergic neurons.

Several other brain areas and cell types can be targeted by IUE at different embryonic ages and with different paddle orientations (Baumgart and Grebe, 2015; Szczurkowska et al., 2016). Augmented specificity in targeting can be achieved by using: (i) intracranial needles instead of extracranial electroporation paddles; (ii) Cre-expressing driver lines and clonal labeling techniques, such as CLoNe (García-Moreno et al., 2014; Vasistha et al., 2015). Further aspects about IUE were recently described in detail (Martínez-Garay et al., 2016).

### Combined Electrophysiology and Light Stimulation *In Vitro*

**Figure 2:**
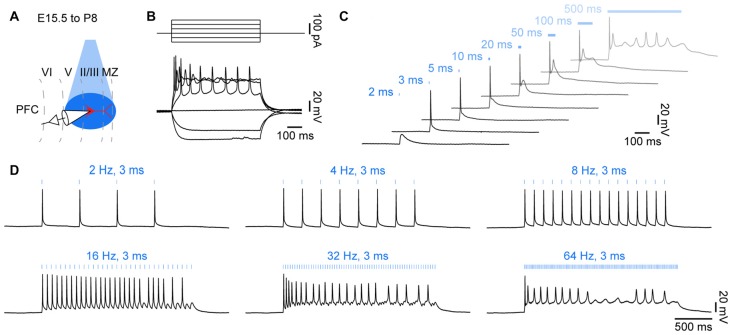
Optogenetic control of action potential firing in neonatal brain slices *in vitro*. **(A)** Diagram illustrating combined light stimulation and whole-cell patch-clamp recordings from layer II/III pyramidal neurons in coronal slices of P8–10 mice transfected with channelrhodopsin 2 double mutant E123T T159C (ChR2(ET/TC)) by IUE at E15.5. **(B)** Voltage responses of a ChR2(ET/TC)-transfected neuron to hyper- and depolarizing current pulses. **(C)** Voltage responses of a ChR2(ET/TC)-transfected neuron to blue light pulses (473 nm, 5.2 mW/mm^2^) of 2–500 ms duration. **(D)** Voltage responses of a ChR2(ET/TC)-transfected neuron to repetitive trains of 3 ms-long light pulses at different frequencies.

To decide whether transfection of neurons of interest by IUE yields sufficient expression of opsins to trigger action potentials already during the neonatal period, we performed whole-cell patch-clamp recordings from transfected neurons in brain slices from P8 to P10 mice *in vitro* (Figure 2A). Pyramidal neurons in the PFC were transfected with the highly-efficient ChR2(ET/TC) under control of the CAG promoter. ChR2(ET/TC) has been reported to have high conductance upon stimulation with blue light, as well as fast kinetics allowing for fast rhythmic stimulation (Berndt et al., 2011). The RFP tDimer2 was co-expressed to identify the transfected neurons.

For whole-cell patch-clamp recordings pups were decapitated, brains were removed carefully within less than a minute, and immediately transferred into ice-cold oxygenated high sucrose artificial cerebrospinal fluid (ACSF) containing (in mM: 228 Sucrose, 2.5 KCl, 1 NaH_2_PO_4_, 26.2 NaHCO_3_, 11 Glucose, 7 MgSO_4_; 320 mOsm, pH 7.2). Coronal sections (300 μm thickness) including the area of interest were sectioned in ice-cold oxygenated high sucrose ACSF and incubated in oxygenated ACSF (in mM: 119 NaCl, 2.5 KCl, 1 NaH_2_PO_4_, 26.2 NaHCO_3_, 11 Glucose, 1.3 MgSO_4_; 320 mOsm, pH 7.2) at 37°C for 45 min before cooling to room temperature for recordings. Fluorescently-labeled neurons were patched under optical control using pulled borosilicate glass capillaries (tip resistance of 4–7 MΩ) filled with potassium-based pipette solution (in mM: 130 K-Gluconate, 10 HEPES, 0.5 EGTA, 4 Mg ATP, 0.3 Na GTP, 8 NaCl; 285 mOsm, pH 7.4). Recordings were controlled with Ephus software in Matlab environment (MathWorks, MA, USA). Capacitance artifacts were minimized using the built-in circuitry of the patch-clamp amplifier (Axopatch 200B, Molecular devices, Sunnyvale, CA, USA). Responses of neurons to hyper- and depolarizing current injections (Figure 2B), as well as to single and repetitive blue light pulses (473 nm, 5.2 mW/mm^2^) of different duration were recorded and digitized at 5 kHz in current-clamp mode.

Data were analyzed offline using custom-written tools in Matlab software. All potentials were corrected for liquid junction potentials with −10 mV for the potassium based electrode solution (Kilb and Luhmann, 2000). The resting membrane potential was measured immediately after obtaining the whole-cell configuration. Active membrane properties and current voltage relationships were assessed by unsupervised analysis of responses to a series of 600 ms long hyper- and depolarizing current pulses. Single and repetitive light pulses of different duration and frequency were used to test for light responsiveness.

Blue light (473 nm, 5.2 mW mm^−2^) pulses of 1 ms durations were sufficient to depolarize the opsin-expressing neurons, whereas longer light pulses reliably evoked single action potentials (Figure 2C). Upon light stimulation for >50 ms, neurons entered a depolarizing plateau potential, similar to the responses to high positive current injections. In contrast, driving opsin expression with synapsin or human elongation factor 1 α promoters was not sufficient to drive action potentials in transfected neurons with similar light power. Thus, expression strength of the highly efficient ChR2(ET/TC) under control of the CAG promoter achieved by IUE is sufficient to optically drive action potentials during neonatal development *in vitro*. Rhythmic stimulation with 3 ms long blue light pulses triggered repetitive firing in opsin expressing neurons (Figure 2D). The fast off-kinetics of ChR2(ET/TC) allows to trigger neuronal firing at frequencies up to 60 Hz in adult neurons (Berndt et al., 2011). Intrinsic neuronal features may explain why pyramidal neurons in the neonatal PFC did not follow rhythmic stimulation at frequencies >16 Hz.

### Combined Electrophysiology and Light Stimulation *In Vivo*

To determine if neuronal spiking can be equally controlled in the intact brain, we combined optogenetic stimulations with recordings of local field potentials (LFP) and multi-unit activity (MUA) from P8 to P10 mice after IUE *in vivo*.

**Figure 3:**
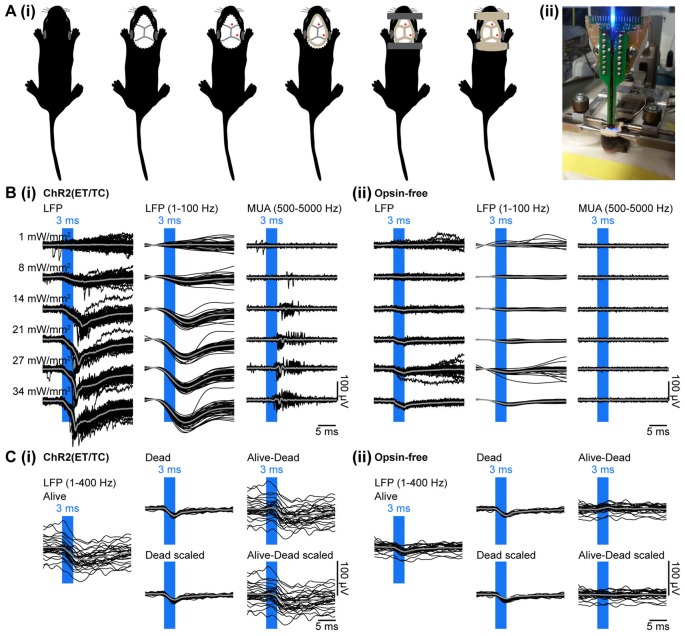
Combined light stimulation and multi-site extracellular recordings from the PFC of neonatal mice *in vivo*. **(Ai)** Diagrams illustrating the surgery for electrophysiology *in vivo* in neonatal mice. The skin is removed from the top of the head and dental cement is applied to strengthen the fragile skull and serve as “glue” for two plastic tubes that enable head fixation. **(ii)** Photograph showing a head-fixed P10 mouse during combined light stimulation and extracellular recordings *in vivo*. **(Bi)** Extracellular recordings (unfiltered local field potentials (LFP), band-pass filtered LFP and MUA) from the PFC of a P10 mouse transfected with ChR2(ET/TC) by IUE at E15.5 in response to light pulses (473 nm, 3 ms) of different intensities. **(ii)** Same as **(i)** for a mouse transfected with an opsin-free construct. **(Ci)** Representative band-pass filtered (1–400 Hz) LFP signals recorded in response to 30 trains of pulsed stimuli (473 nm, 3 ms, 14 mW/mm^2^) before (Alive) and after a lethal injection of ketamine-xylazine (Dead, Dead scaled) from a neonatal mouse transfected by IUE with ChR2(ET/TC). The LFP trace after removal of photoelectric artifacts was obtained by subtraction of the scaled artifact (Alive-Dead scaled). **(ii)** Same as **(i)** for an opsin-free mouse. For **(B,C)** individual traces are shown in black, averages are shown in gray.

The surgery for *in vivo* electrophysiology was done as previously described (Hanganu et al., 2006; Brockmann et al., 2011). Newborn mice have a very fragile skull and their ear channels are closed. This prevents head fixation as typically used for acute extracellular recordings in juvenile or adult rodents. Neonatal mice were injected i.p. with urethane (1 mg/g body weight; Sigma-Aldrich, St. Louis, MO, USA) prior to surgery to achieve stable, long-lasting anesthesia during the recording. Under isoflurane anesthesia (induction: 5%, maintenance: 2.5%) the head of the pup was fixed into a stereotaxic apparatus using two plastic bars mounted on the nasal and occipital bones with dental cement (Figure 3Ai). Interestingly, it is possible to observe the RFP expression through the bone after removal of the skin using a portable fluorescent flashlight for all ages tested (P8–25). The bone above the medial PFC (0.5 mm anterior to Bregma, 0.1 mm right to the midline for layer II/III, 0.5 mm for layer V/VI) or the HP (2.0 mm posterior to Bregma, 0.75 mm right to the midline for dorsal HP; 3.5 mm posterior to Bregma, 3.5 mm right to the midline for intermediate HP) was carefully removed by drilling a hole of <0.5 mm in diameter. After a 10–20 min recovery period on a heating blanket, multi-site optoelectrodes (NeuroNexus, Ann Arbor, MI, USA) were inserted 2–2.4 mm deep into the PFC perpendicular to the skull surface or 1.5–1.8 mm deep into the HP with manual micromanipulators (Figure 3Aii). One-shank multi-site optoelectrodes contained 1 × 16 recordings sites (0.4–0.8 MΩ impedance, 50 or 100 μm spacing) aligned with an optical fiber (105 μm diameter) ending 200 μm above the top recording site. A silver wire was inserted into the cerebellum and served as reference electrode. Extracellular signals were band-pass filtered (0.1–9000 Hz) and digitized (32 kHz) with a multi-channel extracellular amplifier (Digital Lynx SX, Neuralynx, Bozeman, MO, USA) and the Cheetah acquisition software (Neuralynx, Bozeman, MO, USA). Pulsed (light on-off), sinusoidal, and ramp (linearly increasing light power) light stimulations were performed with an arduino uno (Arduino, Italy) controlled diode laser (473 nm, Omicron, Austria). After recordings, animals were transcardially perfused, brains were sectioned coronally, and wide field fluorescence images were acquired to reconstruct the recording electrode position in relation to the neurons transfected by IUE. Data were analyzed offline using custom-written tools in Matlab software. Data were band-pass filtered (500–5000 Hz) to analyze MUA and low-pass (<1500 Hz) filtered using a third order Butterworth filter before downsampling to 3.2 kHz to analyze LFP, before further band-pass filtering (1–100 or 1–400 Hz). All filtering procedures were performed in a manner preserving phase information. MUA was detected as the peak of negative deflections greater than five times the standard deviation of filtered signals. Time-frequency plots were calculated by transforming the data using Morlet continuous wavelet.

Multi-site optoelectrodes allowed simultaneous stimulation by light and recording of the neuronal activity. To validate that neuronal spiking can be reliable triggered in neonatal mice targeted by IUE, light pulses of 3 ms duration were delivered at different light intensities. To control for possible heating and photoelectric artifacts, stimulation was performed in mice transfected with opsin-containing (Figure 3Bi) and opsin-free constructs (Figure 3Bii). Recordings in opsin-free mice did not show any change in MUA upon light stimulation, indicating the absence of induced or suppressed neuronal activity due to potential tissue heating. Modeling of heat propagation in neuronal tissue confirmed low levels of tissue heating for our stimulation parameters (Stujenske et al., 2015; Bitzenhofer et al., 2017). In opsin-expressing mice, light stimulation caused augmented MUA, both shortly after the stimulus, as result of firing of opsin-expressing neurons and at delayed time windows after the stimulus, as result of the activation of neurons downstream to the opsin-expressing neurons.

To ensure that the detected spikes represent neuronal firing and not photoelectrical artifacts that can be evoked by photons hitting the recording sites (Cardin et al., 2010), we assessed the response to single light pulses in more detail. Photoelectric artifacts typically occur immediately after abrupt changes in light intensity (Cardin et al., 2010). No fast transitions were observed at the start and the end of the light pulse in the band-pass filtered (500–5000 Hz) signal used for spike detection (Figure 3B), indicating that MUA was not contaminated by photoelectric artifacts. This conclusion was confirmed by the typical delay of spiking (4–8 ms) after the start of the light pulse. Thus, transfection of opsins by IUE yields sufficient expression to trigger neuronal firing in neonatal mice *in vivo*.

In contrast to MUA, LFP recordings were artifactually altered by light. Two types of artifacts were detected. First, the photoelectric artifacts were identified as short small-amplitude deflections in opsin-free animals (Figure 3Bii). Second, large amplitude long-lasting negative deflections were present only in opsin-expressing animals (Figure 3Bi). They presumably represent physiological extracellular current sinks that are created by the flow of sodium ions into neurons due to the simultaneous opening of the light-gated channelrhodopsins expressed in the membranes of neurons surrounding the recording sites upon light stimulation. This is corroborated by the similarity of their timing (start 2 ms after stimulation onset and last for 8–12 ms) to the kinetics of ChR2(ET/TC). These slow negative deflections of large amplitude disappeared after a lethal injection of ketamine-xylazine, whereas photoelectric artifacts persisted (Figure 3Ci). The photoelectric artifacts were recorded post-mortem, filtered (1–400 Hz), averaged, scaled to the immediate downstroke (0–1.5 ms after light onset) of the alive recordings and subtracted from the alive recordings (Figures 3Ci,Cii). Thereby, photoelectric artifacts can be removed from the recordings.

## Results

### Different Light Stimulation Patterns Are Instrumental for the Characterization of Neonatal Firing

The protocols described above were used to manipulate the firing and network activity of different brain areas during neonatal development (Bitzenhofer et al., 2017). For this, we tested three light stimulation patterns and recorded the neuronal and network responses in the PFC and HP of neonatal mice *in vivo*: (i) trains of repetitive short square pulses; (ii) sinusoidal; and (iii) ramp stimulations (Adesnik and Scanziani, 2010; Cardin et al., 2010; Stark et al., 2013).

**Figure 4:**
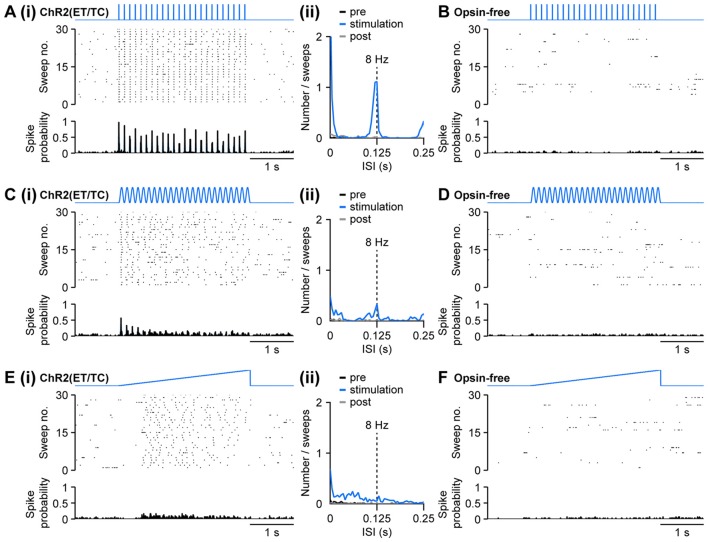
Optogenetic activation of pyramidal neurons in the neonatal PFC by different patterns of light stimulation *in vivo*. **(Ai)** Representative raster plot and spike probability histogram displaying the firing of ChR2(ET/TC) transfected layer II/III pyramidal neurons in response to 30 sweeps of repetitive light pulses (473 nm, 3 ms, 14 mW/mm^2^) at 8 Hz recorded from a neonatal mouse after IUE at E15.5. **(ii)** Corresponding inter-spike interval (ISI) histogram averaged for 3 s before light stimulation (pre, black), 3 s during ramp stimulation (stimulation, blue), and 3 s after light stimulation (post, gray). **(B)** Same as **(Ai)** for an opsin-free control animal. **(Ci,ii,D)** Same as **(Ai,ii,B)** for sinusoidal light stimulation. **(Ei,ii,F)** Same as **(Ai,ii,B)** for ramp light stimulation.

The most commonly used stimulation pattern is a train of short square pulses repeated at a specific frequency. This stimulation pattern drives opsin-expressing neurons to synchronously fire at the stimulated frequency. When applied to glutamatergic layer II/III neurons of the neonatal PFC transfected with ChR2(ET/TC) the stimulation (30 sweeps of rhythmic 3 ms long light pulses at 8 Hz) led to reliable and rhythmic firing (Figures 4Ai,ii). No MUA response to rhythmic light pulses was observed in control mice after IUE with opsin-free plasmids, encoding only for the RFP tDimer2 (Figure 4B).

Similar to repetitive light pulses, stimulation with sinusoidally modulated light power at 8 Hz triggered firing in opsin-expressing, but not in opsin-free animals (Figures 4Ci,D). In contrast to stimulation by trains of light pulses, spiking was less precisely timed for sinusoidal stimulations and did not follow the stimulation reliably. Still, a distinct, but broader peak was observed in the inter-spike interval (ISI) histogram at the stimulation frequency of 8 Hz (Figure 4Cii). In contrast to pulsed stimulation, the precision of light-induced firing decreased towards the end of the stimulation.

Similar to the pulsed and sinusoidal stimulations, ramp stimulation induced spiking in mice electroporated with opsins, but not in opsin-free controls (Figures 4Ei,F). In contrast to the repetitive stimulations at precisely defined frequency, ramp stimulation, with linearly increasing light power, does not time the neuronal firing to a set rhythm. It rather enables a more physiological activation and neurons spike at their preferred frequency, if any, when a certain level of depolarization has been reached. The preferred frequency can reflect intrinsic neuronal mechanisms, but also features of local networks. For example, ramp stimulation of pyramidal neurons in layer II/III of neonatal PFC led to a broad peak between 30 ms and 80 ms, corresponding to the beta frequency band of 12–30 Hz, in the spiking histograms (Figure 4Eii), whereas a preferred firing frequency upon light stimulation cannot be detected in layer V/VI pyramidal neurons (Bitzenhofer et al., 2017).

Thus, different light stimulations can be applied to induce firing of neonatal neurons transfected with opsins by IUE. They allow not only timing of the activity within developing neuronal networks, but also the dissection of intrinsic rhythms of activation.

### Different Light Stimulation Patterns Are Instrumental for the Interrogation of Developing Circuits

To assess the effects of distinct patterns of light stimulation on the oscillatory activity of developing neuronal networks, we simultaneously stimulated with light and recorded the LFP in layer II/III of the medial PFC of opsin-expressing and opsin-free P8–10 mice after IUE at E15.5.

**Figure 5:**
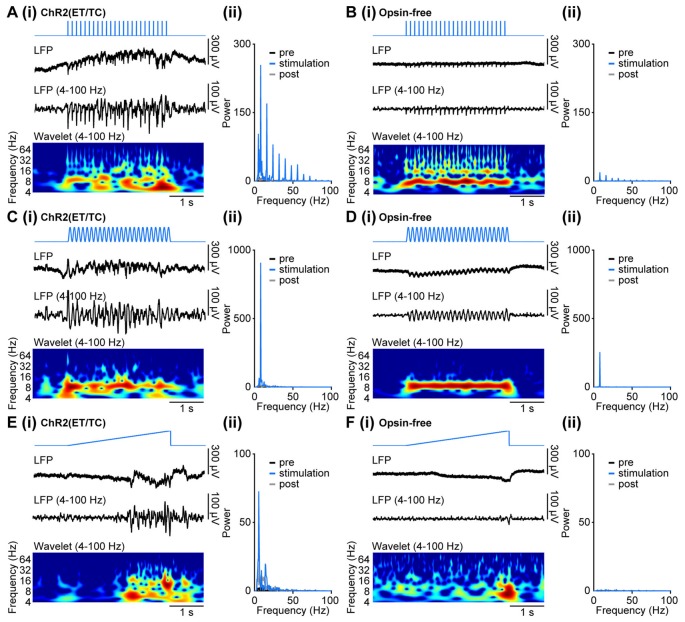
Generation of coordinated network activity in the neonatal PFC as result of different patterns of light stimulation *in vivo*. **(Ai)** Discontinuous oscillatory activity displayed together with the corresponding color-coded wavelet spectrum at identical time scale recorded in response to repetitive light pulses (473 nm, 3 ms, 14 mW/mm^2^) at 8 Hz from a P10 mouse with layer II/III pyramidal neurons transfected by IUE at E15.5 with ChR2(ET/TC). **(ii)** Plots displaying the corresponding power spectrum for 3 s before light stimulation (pre, black), 3 s during ramp stimulation (stimulation, blue), and 3 s after light stimulation (post, gray). **(Bi,ii)** Same as **(Ai,ii)** for a P10 mouse transfected by the same procedure with an opsin-free construct. **(Ci,ii,Di,ii)** Same as **(Ai,ii,Bi,ii)** for sinusoidal light stimulation. **(Ei,ii,Fi,ii)** Same as **(Ai,ii,Bi,ii)** for ramp light stimulation.

In contrast to the light-induced spiking, the band-pass (4–100 Hz) filtered LFP was profoundly affected by photoelectric artifacts when trains of light pulses were used (Figures 3B,C). The repetitive stimulation induced large negative deflections detectable in the LFP of opsin-expressing mice (Figure 5Ai). While opsin-free animals showed only the photoelectric artifact (Figure 5Bi), three distinct effects were detected in opsin-expressing pups: (i) immediate photoelectric artifacts when light was switched on and off; (ii) large magnitude negative deflections with slower kinetics due to the simultaneous opening of the light-gated channelrhodopsins and the resulting flow of positively charged ions into neurons; (iii) and coordinated activity within local networks resulting from light-induced spiking, which has been described above. The large deflections (ii) contaminated the time resolved frequency spectra and the power spectra not only at the frequency of stimulation, but also at the corresponding higher frequency harmonics due to their rhythmic, non-sinusoidal shape (Figures 5Aii,Bii). The mixed effects of the deflections precluded a quantitative analysis of light-induced network activity during the stimulation, despite the fact that the pure photoelectric artifacts can be removed (see “Materials and Methods” Section).

Similarly, sinusoidal light stimulations resulted in a mixture of photoelectric artifacts, large light-induced deflections, and light-induced coordinated activity in the local networks of opsin-expressing animals (Figures 5Ci,Di). Photoelectric artifacts are detectable as peaks of oscillatory power at the set stimulation frequency in both opsin-expressing and opsin-free pups (Figures 5Cii,Dii). The small broader power peak around the stimulation frequency was detected only in opsin-expressing animals and most likely reflects induced coordinated activity. The sinusoidal shape of the stimulation artifact restricts the contamination of the power spectrum to the stimulation frequency, without the induction of higher frequency harmonics. Similar to pulsed stimulation, the quantification of light-induced coordinated network activity cannot be achieved during stimulation, but only by comparison of time windows shortly before and after stimulation.

In contrast to pulsed and sinusoidal stimulations, ramp stimulation did not cause rhythmic artifacts, due to the slow change in light power (Figures 5Ei,Fi). Only the abrupt switch from light on to off at the end of the ramp stimulation led to a small photoelectric artifact detectable in the LFP. Therefore, quantification of induced oscillatory activity during the stimulation was possible. For example, investigation of LFP effects after ramp stimulation of layer II/III pyramidal neurons of the medial PFC in P8–10 opsin-expressing mice showed that prominent beta band network oscillations can be induced by light (Figure 5Eii; Bitzenhofer et al., 2017). They correspond to the preferred firing of neurons within similar frequency range upon ramp stimulation (Figure 4Eii). In opsin-free animals, light stimulation did not induce oscillatory activity and the power spectrum remained unchanged (Figure 5Fii).

Thus, coordinated patterns of oscillatory activity in neonatal mice can be induced by light, yet depending on the stimulation patterns, they might be contaminated by photoelectric and physiological artifacts. These artifacts are negligible for ramp stimulation, making this pattern the method of choice for the investigation of light-induced effects on oscillatory network activity.

## Discussion

Controlling the activity of specific neurons at fast time scales by optogenetics has proven successful to elucidate the contribution of identified neuronal populations to synchronous network activity in the adult brain (Cardin et al., 2009; Adesnik and Scanziani, 2010; Tye and Deisseroth, 2012). Similar investigations in developing neuronal networks have been hampered by difficulties to reliably and specifically express opsins during neonatal development. Here, we describe a protocol that allows the application of optogenetics for the interrogation of neuronal networks during first postnatal weeks in mice, as shown for the neonatal PFC and HP.

The effective and selective expression of opsins in a subset of neurons during early life is the prerequisite for the optogenetic interrogation of the cellular interactions and their contribution to coordinated activity patterns within developing neuronal networks. Similar early targeting of neurons was required for the study of the migration of cortical neurons during embryonic and early postnatal development (Tabata and Nakajima, 2001; Borrell et al., 2005). For this, fluorescent markers were expressed early in cellular populations of interest by IUE. In the protocol described above, we used the advantages of the early expression onset achieved with this technique, as exemplified by the reliable and stable expression of opsins in specific populations of pyramidal cells in the medial PFC and the HP during early postnatal development.

Neuronal precursors, generating different neuronal populations that are intermixed in neuronal networks after migration, are spatially and temporally separated during embryonic development (Wonders and Anderson, 2006; Hoerder-Suabedissen and Molnar, 2015). The spatially separated generation of glutamatergic and GABAergic neurons prevents the need of cell type specific promoters to achieve cell-type specific expression with IUE (Wonders and Anderson, 2006; Hoerder-Suabedissen and Molnar, 2015). Furthermore, the targeting confined to specific embryonic stages and to specific groups of precursor cells in the ventricular zone enables to achieve expression that is restricted to distinct brain regions, such as different areas of the neocortex, HP, cerebellum, striatum, thalamus or hypothalamus (Baumgart and Grebe, 2015; Szczurkowska et al., 2016). The age-dependent formation of cortical layers in an inside-out sequence allows targeting of principal neurons in a layer-confined pattern, when IUE is performed at the corresponding embryonic day (Taniguchi et al., 2012). We exemplified this by separately addressing the upper (layer II/III) and deeper layers (layer V/VI) of the PFC. Therefore, expression in specific neuronal populations, that is restricted to specific brain areas, and even to specific cortical layers, can be achieved by IUE, without the need of population specific promoters. Ubiquitous promoters can be used to yield stable expression during development and comparable expression in different types of neurons. This is one of the major advantages of IUE when compared to targeting by viral injections and genetic mouse lines. Promoters specific for certain types of neurons are often unspecific during early development, or do not drive constant expression over time (Sánchez et al., 1992; Kwakowsky et al., 2007; Wang et al., 2013). IUE is suitable to investigate neuronal networks over long time periods, since it ensures stable expression of opsins throughout the development and at adulthood (Adesnik and Scanziani, 2010). Moreover, opsin transfection using IUE did not affect the patterns of neuronal activity when compared to those from non-electroporated and opsin-free neonatal mice (Bitzenhofer et al., 2017).

The highly efficient channelrhodopsin 2 mutant ET/TC causes large photocurrents and has fast off kinetics. Transfection by IUE under the control of the ubiquitous CAG promoter yielded sufficient expression to precisely control the generation of action potentials in neonatal mice by light, both under *in vitro* and *in vivo* conditions. We compared the effects of different stimulation patterns (repetitive light pulses, sinusoidally and ramp modulated light intensity) on extracellularly recorded spiking and oscillatory activity in neonatal mice after area-, layer- and cell-type-specific transfection with opsins by IUE. All stimulation patterns induced reliable spiking that was largely unaffected by photoelectric artifacts, indicating the general feasibility of integrated local stimulation and recording within the same volume. Pulsed stimulations induced a highly synchronous spiking reliably following the stimulation frequency. Similarly, sinusoidal stimulation also induced synchronous spiking activity although with higher variability over the stimulation period. The entrainment following pulsed stimulation is possibly excessive with spiking activity tightly following the stimulation frequency. In contrast to spiking in a defined stimulation frequency, ramp stimulation does not impose a specific frequency on neuronal firing, but drives neuronal networks to spike at an intrinsic or preferred rhythm (Adesnik and Scanziani, 2010; Bitzenhofer et al., 2017). Thus, each stimulation type induces a different type of spiking activity and synchrony in the network and can be adjusted to the experimental needs.

Photoelectric artifacts and large voltage deflections induced by repetitive pulsed and sinusoidal stimulations precluded a quantitative analysis of light-induced oscillatory activity in developing circuits. Solely the network entrainment before and after the stimulation time window can be compared. The size of the photoelectric artifact is highly dependent on the amount of light hitting the metallic recording site. It can be reduced by angling the light fiber in such that the light cone does not emit directly to the recording site, or by recording outside of the illuminated area. Alternatively, recordings with glass electrodes are not affected by photoelectric artifacts (Cardin et al., 2010). Sinusoidal stimulation is advantageous when investigating frequencies of the LFP outside the frequency used to stimulate, due to minimal induction of harmonics in the power spectrum (Akam et al., 2012). The slow change in light intensity during ramp light stimulation did not induce a photoelectric artifact or rhythmic physiological current sinks due to the simultaneous opening of channelrhodopsins, enabling to quantify induced oscillatory activity in the local network during optical stimulation. We recently used this stimulation pattern to provide direct evidence for the contribution of layer II/III, but not layer V/VI, pyramidal cells to beta-gamma oscillatory activity in the medial PFC in neonatal mice (Bitzenhofer et al., 2017).

The protocol described here opens new perspectives for the interrogation of neuronal networks and the elucidation of cellular interactions during brain development. The method can be combined with designer receptor exclusively activated by designer drugs (DREADDs; Ishii et al., 2015), calcium-indicators (Gee et al., 2015), or region specific gene manipulation (Niwa et al., 2010; Page et al., 2017) to get deeper insights into the mechanisms of circuit wiring. Furthermore, chronic optogenetic manipulation of defined neuronal populations and oscillatory rhythms during specific developmental periods will help to elucidate the role of early network activity for behavioral performance at adulthood under physiological and pathological conditions.

## Author Contributions

ILH-O designed the experiments. SHB and JA developed the protocol and carried out the experiments, SHB and JA analyzed the data. ILH-O, SHB and JA interpreted the data and wrote the manuscript.

## Conflict of Interest Statement

The authors declare that the research was conducted in the absence of any commercial or financial relationships that could be construed as a potential conflict of interest.

## Abbreviations

ACSF: artificial cerebrospinal fluid
CAG: cytomegalovirus enhancer fused to chicken beta-actin
CaMKII: Ca^2+^/calmodulin-dependent protein kinase II
ChR2(ET/TC): channelrhodopsin 2 double mutant E123T T159C
DREADDs: designer receptor exclusively activated by designer drugs
E: embryonic day
GABA: gamma-aminobutyric acid
HP: hippocampus
ISI: inter-spike interval
IUE: *in utero* electroporation
LFP: local field potential
MUA: multi unit activity
P: postnatal day
PBS: phosphate buffered saline
PFC: prefrontal cortex
RFP: red fluorescent protein.
